# Clear-Cell Carcinoma of the Ovary with Bilateral Breast Metastases

**DOI:** 10.1155/2019/8013913

**Published:** 2019-04-28

**Authors:** Yehuda Galili, Meghan Lytle, Jonathan Bartolomei, Kataria Amandeep, Nichole Allen, S. J. Carlan, Mario Madruga

**Affiliations:** ^1^Department of Internal Medicine, Orlando Regional Healthcare, Orlando, Florida, USA; ^2^Florida State University College of Medicine, Tallahassee, FL, USA; ^3^Department of Pathology, Orlando Regional Healthcare, Orlando, Florida, USA; ^4^Division of Academic Affairs and Research, Orlando Regional Healthcare, Orlando, Florida, USA

## Abstract

Ovarian clear-cell carcinoma is an uncommon subtype of epithelial ovarian carcinoma. It carries a generally poor prognosis because of its resistance to standard treatment and metastatic spread to vital organs. Metastasis to the breast is rare and bilateral breast metastasis is unreported. A 61-year-old white female with a 5-year status poststandard therapy for stage IC clear-cell ovarian carcinoma presented with widespread metastasis. Tissue analysis revealed ovarian cancer metastasis to the breasts bilaterally. Clinical awareness of this metastatic potential is important when staging and developing a treatment plan for patients with ovarian clear-cell cancer.

## 1. Introduction

Ovarian cancers are the second most common female gynecologic cancer and are the leading cause of death from gynecologic malignancy in the United States [1]. They commonly arise in women in the fifth to seventh decade of life [2]. Clinical manifestations commonly include abdominal and pelvic pain with bloating, distention, urinary urgency and frequency, and unintended weight loss in the presence of tenderness to palpation and an adnexal mass on bimanual examination. Risk factors for the development of ovarian cancer include early menarche, late menopause, nulliparity, and infertility. Additional risk factors include the BRCA1 and BRCA2 (breast cancer 1 and 2 gene) mutations, as well as Lynch syndrome.

Epithelial ovarian cancers cover a majority of the malignant ovarian cancers and are classified based on histologic morphology [2]. Ovarian clear-cell carcinoma (OCCC) is of the epithelial subtype, occurring in only 3% of ovarian cancers with an increased prevalence in Japanese women [2–4]. This specific type of ovarian malignancy distinguishes itself from others in the epithelial subtype and carries a generally poor prognosis because of its resistance to standard treatment with platinum and taxane-based agents [5, 6].

Fourteen percent of patients with the clear-cell subtype present with lymph node metastasis during stages I-II of the disease course, commonly affecting the pelvic and para-aortic lymph nodes. However, hematogenous spread at the time of diagnosis is not common. Later in the disease course, patients can develop metastatic spread to vital organs, possibly as a result of leakage or rupture of cells from the intraperitoneal mass, occurring in as many as 38% of patients with stage IV disease [7]. The most common sites of metastasis are the lung and liver during advanced stages of the disease and these patients often present with ascites or pleural effusion [8].

In general, metastasis to the breast from an extra mammary neoplasm is extremely rare, occurring in less than 1% of cases [9]. Furthermore, reports of metastatic spread of a primary OCCC to the breast are not well documented, with only 39 reported cases in current literature [10]. We present a patient who was diagnosed with bilateral breast metastases resulting from an OCCC primary tumor.

## 2. Case

**Figure 1:**
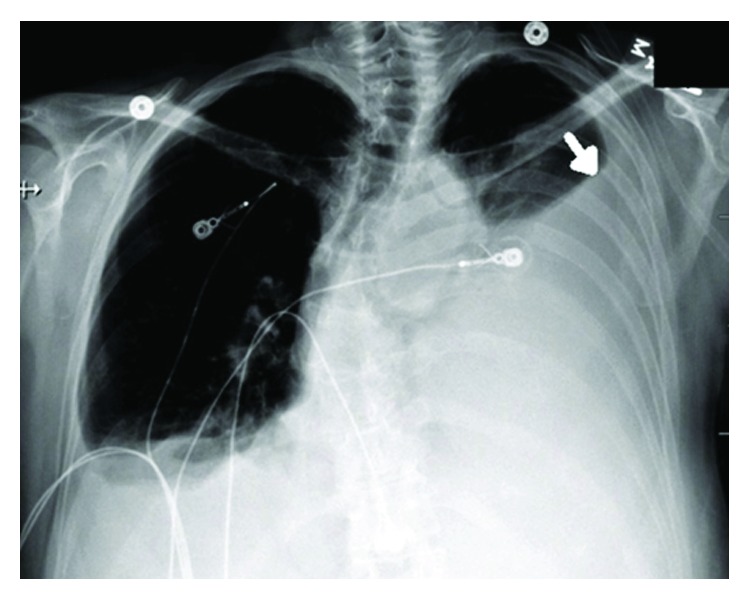
PA chest radiograph showing a large left-sided pleural effusion (arrow).

**Figure 2:**
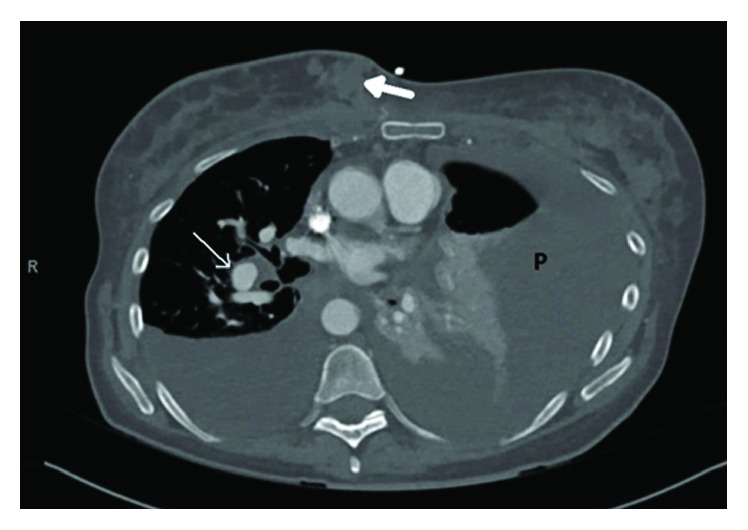
Computed tomography with angiography showing a right medial breast mass (closed white arrow) with mediastinal (open white arrow) and axillary lymphadenopathy. Large effusions greater on the left (P).

**Figure 3:**
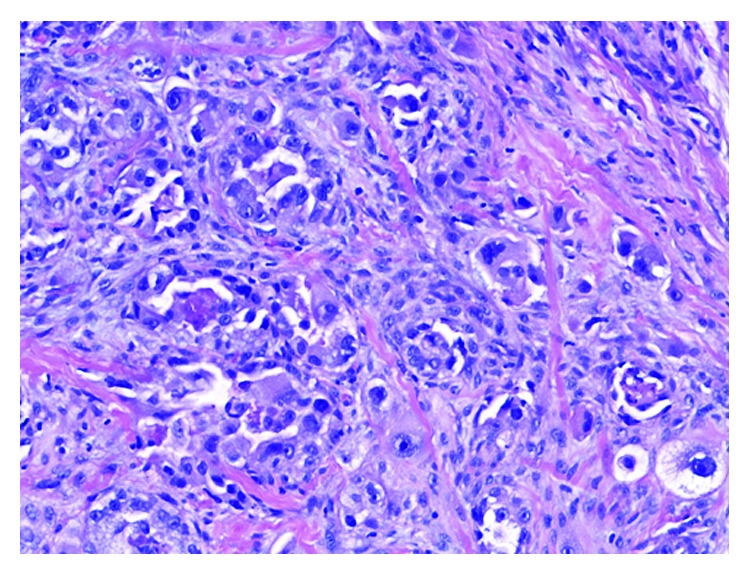
Hematoxylin and eosin stain showing invasive carcinoma consisting of pleomorphic tumor cells with vacuolated and eosinophilic cytoplasm and prominent nucleoli.

**Figure 4:**
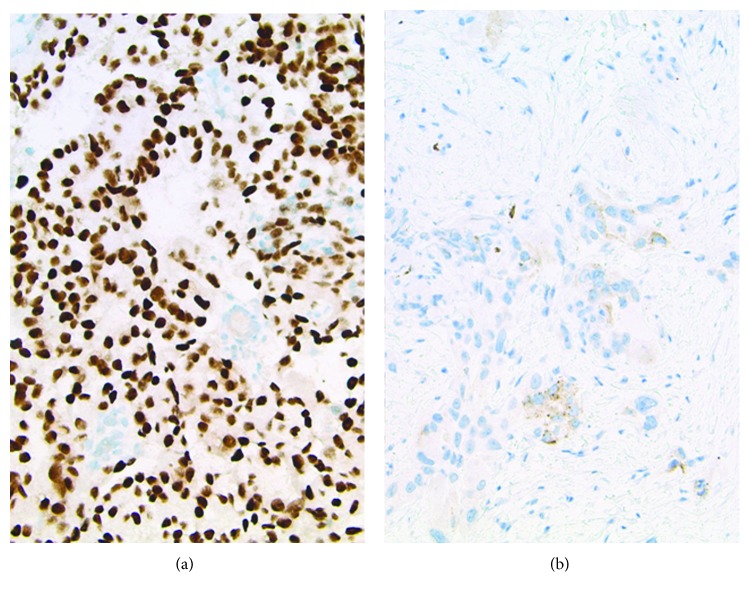
(a) PAX-8: strong, diffuse nuclear staining within tumor cells is consistent with a Mullerian primary and militates against lung primary. (b) Napsin-A: focal granular cytoplasmic staining, consistent with either lung or clear-cell Mullerian-type adenocarcinoma.

This is a 61-year-old Caucasian female with significant past medical history of ovarian cancer complaining of shortness of breath for several weeks. Five years prior, the patient was diagnosed with stage IC clear-cell ovarian carcinoma and had undergone robotic-assisted laparoscopic hysterectomy, bilateral salpingo-oophorectomy, omentectomy, pelvic and periaortic lymphadenectomy, and 3 cycles of carboplatin and paclitaxel intravenous and intraperitoneal with no evidence of disease on imaging. Her last cancer antigen 125 (CA 125) level was 8. Unfortunately, she lost a follow-up with her oncologist until this hospitalization. On admission, she stated symptoms started 2 weeks prior, were worse on exertion, and were associated with a dry cough and 10 pounds of unintentional weight loss. She denied fevers, chills, night sweats, chest or abdominal pain, diarrhea, or constipation. Reproductive history was significant for 2 full-term vaginal deliveries with 2 living sons, menarche at 12 years old and menopause at 56 years old. Her family history was significant for her paternal grandmother with breast cancer in her 60s, but no history of gynecologic or colon cancer. She denied ever using tobacco, alcohol, or illicit drugs. Upon further questioning, she stated that over the past 6-8 weeks, she noticed a tender lump in her right breast. On admission, vital signs were significant for oxygen saturation of 92% on 4-liter nasal cannula. On physical exam, she was an ill-appearing thin female in mild distress secondary to shortness of breath. Lung examination yielded decreased breath sounds bilaterally and diminished at the bases. Breast examination yielded a firm right-sided chest mass just right of midline measuring 8 × 4 centimeters. Complete blood count and metabolic panel were unremarkable. Chest radiography showed a large left-sided and small right-sided pleural effusions (Figure 1). Computed tomography (CT) with angiography revealed a right medial breast mass, mediastinal and axillary lymphadenopathy, and bilateral effusions, greater on the left (Figure 2). CT abdomen and pelvis showed a small amount of ascites and mesenteric metastases. An ultrasound-guided thoracentesis was performed which aspirated 1.1 liters of clear yellow fluid from the left pleural space. The fluid was sent for cell culture and cytology. The fluid was consistent with metastatic clear-cell carcinoma of the ovary. Ultrasound of the breasts showed a right-sided dominant malignant appearing lesion in medial breast with axillary adenopathy and left-sided multiple malignant appearing breast and pectoral lesions with left axillary adenopathy. She underwent ultrasound-guided biopsy of left and right breast lesions as well as left axillary lymph node. Pathology from both breast tissue samples revealed Mullerian epithelial primary, consistent with metastatic clear-cell carcinoma of the ovary (Figures 3 and 4). Repeat CA-125 was strongly positive at 318. She was referred to gynecology oncology service. Upon follow-up, she was started on chemotherapy with docetaxel, carboplatin, and bevacizumab. Next-generation sequencing was ordered. Radiation oncology was consulted. Genetic counseling and testing were recommended. Her cancer was re-staged as stage IV clear-cell carcinoma of the ovary with metastases to the bilateral breasts, axillary lymph nodes, and abdomen.

## 3. Discussion

Ovarian clear-cell carcinoma accounts for less than 10 percent of all ovarian carcinomas in North America [11, 12]. It appears to account for a larger portion of ovarian cancers in East Asia for unclear reasons [13]. In addition, OCCC with metastasis to bilateral breasts has not been reported to date. We performed a MEDLINE search of the English language literature from January 1, 1966, to December 31, 2017, using the keywords “clear cell carcinoma,” “ovary,” “metastasis/metastatic,” “bilateral,” and “breast.” We could find, however, no cases of ovarian clear-cell carcinoma that had metastasized to bilateral breasts.

The clinical presentation of OCCC with metastasis appears to vary based on the histological subtype and disease burden [13, 14]. When breast metastasis does occur, it most commonly presents as a solitary mass that could be clinically mistaken for primary breast malignancy. Though infrequent, it may also present with lesions that mimic inflammatory carcinoma of the breast, as suggested in one report of metastatic OCCC. Generally, breast metastasis is diagnosed an average of two years after the initial diagnosis of a primary ovarian source [10]. Despite positive ultrasound findings for malignancy seen in right breast tissue of our patient, findings may be equivocal as metastatic lesions may resemble benign or malignant disease [9]. In the setting of high clinical suspicion, direct comparison between breast fine needle aspirate cytology and the original primary ovarian tumor should confirm the diagnosis [15], as evidenced by Mullerian epithelial cells in both breast tissue and pleural fluid in our patient.

OCCC often presents at an early stage (stage I or II) and has a relatively good prognosis. However, presentation with advanced disease stage or recurrence has a worse prognosis than the more common subtypes serous and endometrioid carcinoma [16]. This is due to the lack of chemosensitivity to platinum-based chemotherapy [17]. The presence of specific molecular characteristics such as PIK3CA (phosphatidylinositol 3-kinase p110*α* catalytic subunit gene) and ARID1a (AT-rich interactive domain-containing protein 1A) mutations, and MET gene amplification have been proposed to be associated with chemoresistance warranting dual inhibitor targeting in advanced stages [18]. Additionally, aberrant expression of CCC- (clear-cell carcinoma-) specific genes via upregulation of HNF-1*β* (hepatocyte nuclear factor) transcription factor expression plays a key role in the pathogenesis of OCCC [19]. Furthermore, OCCC displays a unique immunophenotype among other epithelial ovarian carcinomas lacking expression of both estrogen receptors and WT-1 (Wilms' tumor suppressor gene) and displays absence of strong diffuse expression of p53 seen in high-grade serous subtype [18], similar to our case. In addition to molecular markers, stage and lymph node status are the only valuable parameters for prognostication, whereas grading based on morphological features is not of prognostic significance [20]. Metastatic OCCC is also associated with an increased risk of vascular thrombosis and paraneoplastic hypercalcemia, which were not evident in our patient [18].

While the pattern of metastasis of OCCC has not been well described due to the paucity of cases, it appears to be more aggressive and less responsive to conventional therapy [10]. A recently published case series describes bone metastases via hematogenous spread at initial presentation. This tends to occur in significantly higher rates in those with clear-cell subtype when compared to high-grade serous carcinoma of the ovary [21]. Secondary breast involvement and widespread disease from ovarian cancer confers a poor prognosis with a survival time range of 18 days to 3.5 years with many patients dying within one year [16]. Metastatic OCCC with breast involvement confers an even worse prognosis with a median survival time of six months.

## 4. Conclusion

Ovarian carcinomas are represented by a number of molecularly distinguishable diseases defined by histological subtype with variable outcomes, patterns of relapse, and response to therapy. Clinicians treating patients with OCCC should be aware of the metastatic potential of this disease, evaluate symptomatic patients accordingly, and encourage close follow-up for monitoring. Ovarian metastasis to the breast represents a diagnostic challenge due to the ambiguous radiographic and clinical findings and warrants further cytological evaluation. Accurate differentiation via pathologic analysis is necessary for diagnostic resolution, especially in patients with a history of primary ovarian carcinoma, as treatment and prognosis differ greatly from primary breast malignancy. Additionally, further characterization of aberrant gene expression and molecular markers may play a role in offering targeted therapy in all stages of OCCC due to chemoresistance to conventional modalities.
